# Identifying ICAM-1 as a Therapeutic Target for Cytokine Storm in Human Macrophages Through Integrative Bioinformatics Approaches

**DOI:** 10.3390/molecules31071111

**Published:** 2026-03-27

**Authors:** Shaojun Chen, Dapeng Wu, Zhe Zheng, Yiyuan Luo, Lihua Zhang

**Affiliations:** 1 Department of Traditional Chinese Medicine, Zhejiang Pharmaceutical University, Ningbo 315000, China; chensj@zjpu.edu.cn (S.C.); luoyy@zjpu.edu.cn (Y.L.); 2 Affiliated Hospital, Nanjing University of Traditional Chinese Medicine, Nanjing 210023, China; wudapeng1515@163.com; 3 School of Pharmacy, Nanjing University of Traditional Chinese Medicine, Nanjing 210023, China; 20230609@njucm.edu.cn; 4 School of Health Management, Zhejiang Pharmaceutical University, Ningbo 315000, China

**Keywords:** cytokine storm, COVID-19, dasatinib, ICAM-1, gene expression signature, weighted gene co-expression network analysis

## Abstract

Excessive macrophage activation is thought to be the primary cause of the cytokine storm that results in severe coronavirus disease 2019 (COVID-19) complications. The underlying mechanisms remain elusive, and more research is needed to find disease-critical genes and develop effective therapies. In this study, we used publicly accessible microarray datasets of cytokine storm in cultured human monocyte-derived macrophages challenged with cytokines, and employed bioinformatics, such as weighted gene co-expression network analysis (WGCNA) and differential expression analysis, to dissect gene expression profiles and identify putative disease-related molecules. Initially, three co-expression modules and related key genes were discovered, which highly correlated to macrophages challenged with cytokines. Then, a preliminary gene expression signature consisting of 203 upregulated and 24 downregulated genes was identified. Next, protein–protein interaction analysis and hub gene identification were used to identify 11 crucial hub genes, namely *tripartite motif-containing 21* (*TRIM21*), *interferon regulatory factor 1* (*IRF1*), *guanylate binding protein 1* (*GBP1*), *transporter associated with antigen processing 1* (*TAP1*), *nuclear myosin I* (*NMI*), *interleukin 15 receptor subunit alpha* (*IL15RA*), *apolipoprotein L1* (*APOL1*), *intercellular adhesion molecule 1* (*ICAM-1*), *protein tyrosine phosphatase non-receptor type 1* (*PTPN1*), *E74-like ETS transcription factor 4* (*ELF4*) and *guanylate binding protein 2* (*GBP2*). Then, the LINCS L1000 characteristic direction signatures search engine (L1000CDS2) was employed for drug repurposing studies. Dasatinib was predicted to be the leading therapeutic compound to perturb the gene signature of cytokine storm in human macrophages. Connectivity Map results suggested that dasatinib may normalize ICAM-1 expression. In addition, the results of molecular docking studies and molecular dynamics simulation revealed that dasatinib may spontaneously interact with ICAM-1 via several key residues and form a relatively stable protein–ligand complex. Overall, this work, based on an analysis of co-expression correlation networks, gene expression signatures and pivotal genes in human macrophages challenged with cytokines, combined with drug repurposing studies, demonstrated that dasatinib may interact with ICAM-1 and could be a potential candidate for cytokine storm. However, due to the limitations of computational approaches, further experimental validation is necessary.

## 1. Introduction

Coronavirus disease 2019 (COVID-19), caused by the SARS-CoV-2 virus, caused a worldwide crisis between 2020 and 2023, with the end of the emergency phase declared in May 2023 [1]. The World Health Organization reported that the pandemic resulted in about 7 million confirmed deaths worldwide, although the actual number of deaths may be much higher. Although COVID-19 has now entered an endemic phase with expected seasonal peaks, it remains a concern worldwide [2]. Aberrant hyperinflammatory responses are the vital clinical detail observed in COVID-19-infected persons [3,4]. Highly increased levels of plasma cytokines, including interleukin 2 (IL2), interleukin 7 (IL7), interleukin 10 (IL10), colony stimulating factor 3 (GCSF, CSF3), interferon gamma-induced protein 10 (IP10), monocyte chemoattractant protein 1 (MCP1), macrophage inflammatory protein 1α (MIP1A,) and tumor necrosis factor α (TNFα) [4], are found in COVID-19 patients. High levels of cytokines and their over-reactivity in the circulation may exceed physiological thresholds, leading to secondary damage and contributing to disease severity and fatality, particularly in individuals with severe COVID-19 [4,5,6,7]. This over-reactivity of cytokines is widely known as cytokine storm, and is a rapidly developing and life-threatening occurrence [5,8].

Macrophages are innate immune cells capable of identifying and reacting to microbial hazards by secreting inflammatory cytokines that efficiently eradicate pathogens and enhance tissue healing [6,9]. These cells play diverse roles in various processes such as development, homeostasis, and the immune response [6,9]. The varying phenotypes and functions of macrophages largely depend on cell plasticity; therefore, macrophages can exert either protective or harmful effects in specific microenvironments [6,9].

Excessive macrophage activation is regarded as the primary cause of the cytokine storm that causes severe COVID-19 complications [10]. Analysis of 93 COVID-19 patient samples showed that alveolar macrophages express many well-known virus-entry molecules such as angiotensin-converting enzyme 2 (ACE2), neuropilin-1 (NRP-1), asialoglycoprotein receptor 1 (ASGR1), and kringle-containing transmembrane protein 1 (KREMNE1) [11]. Autopsy analysis of two COVID-19 patients revealed that the spike protein, a key structural molecule of the SARS-CoV-2 virus, can directly bind to lung macrophages but not to T lymphocytes [12]. Single-cell atlases of autopsy tissue revealed that lung macrophages were increased and enriched for SARS-CoV-2 RNA [13]. Plasmacytoid dendritic cells produce IFN-α, which results in robust inflammatory activation in lung macrophages from severe COVID-19 patients [14]. In addition, pro-inflammatory monocyte-derived macrophages were notably rich in the bronchoalveolar lavage fluid from critical COVID-19 patients [15]. Exposure of human monocyte-derived macrophages to SARS-CoV-2 resulted in high expression of pro-inflammatory cytokines and associated molecules, while simultaneously attenuating type I interferon activity; this finding shows that monocyte-derived macrophages trigger inflammatory cascades and secondary tissue damage [16]. Therefore, targeting macrophage-induced cytokine storm is a potential therapeutic way to combat severe COVID-19 [2,8].

**Figure 1:**
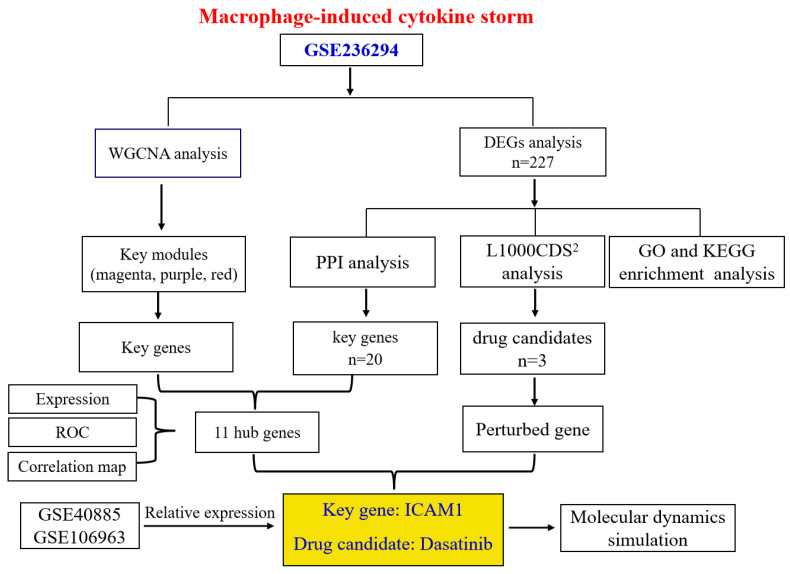
Schematic workflow of bioinformatics analysis to identify key genes and candidate drugs in macrophage-induced cytokine storm.

Accordingly, this study aimed to utilize extensive genetic data to gain a deeper comprehension of the disease-critical genes underlying macrophage-induced cytokine storm and to propose candidate compounds that could likely cure the disorder. Gene expression datasets were retrieved from the publicly accessible Gene Expression Omnibus (GEO) database of the National Center for Biotechnology Information. Using these datasets, co-expression correlation networks, differentially expressed genes (DEGs) and crucial genetic markers were identified using several bioinformatics tools. Furthermore, we looked for small-molecule compounds that could perturb the cytokine storm-related aberrant gene changes observed in macrophages challenged with cytokines. The workflow used in the present study is visualized in Figure 1.

## 2. Results

### 2.1. Screening of Key Genes Based on Gene Modules of WGCNA

**Figure 2:**
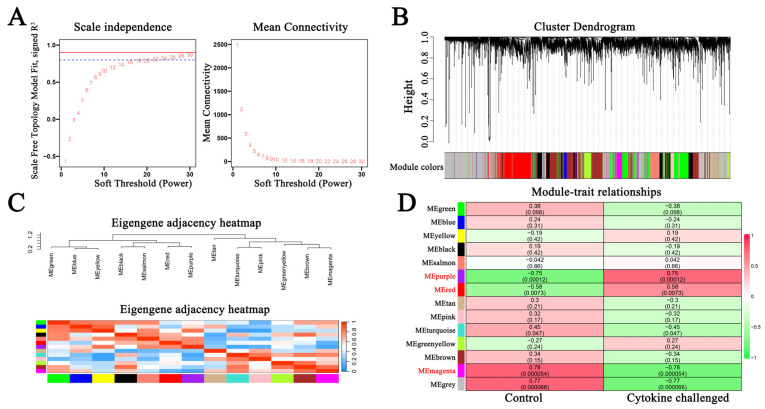
Weight co-expression network analysis (WGCNA). (**A**) Soft threshold selection; (**B**) co-expression similarity of all gene modules based on the clustering dendrogram. Each vertical line stands for a gene, branches for co-expressed genes. (**C**) An eigengene adjacency heatmap; (**D**) module correlations between cytokine-challenged and control groups. Thirteen modules were identified. Upper values in each cell represent module–trait correlation coefficients, while lower values represent the corresponding *p*-values.

The WGCNA method was utilized to discover key gene modules that correlated to macrophages challenged with cytokines. After the raw data of GSE236294 were cleaned and pretreated, the top 8000 genes were selected and subsequently used to form a weighted co-expression scale-free network with a soft threshold power of 22 (Figure 2A). Then, the dynamic tree-cutting tool (cutting height was set at 0.25) was utilized to identify genes that exhibit comparable expression patterns. The vertical black lines in the upper section (depicted in Figure 2B) signify individual modules, while the height (the tree-cutting line) indicates the degree of similarity among the genes. The colored bar in the lower section (Figure 2B) designates the specific colors allocated to each individual module.

Our results show that 13 distinct modules were identified for GSE236294 (Figure 2C). The correlation between the magenta, purple or red gene modules and the cytokine treatment group exceeded 0.5 and the *p*-value was below 0.01 (Figure 2D). These results indicate that the three gene modules may be involved in the cytokine storm in macrophages. In accordance with the criteria of gene significance > 0.5 and module membership > 0.5, there were 238, 204 and 229 genes identified within the magenta, purple and red gene modules, respectively (Supplementary Tables S1–S3).

### 2.2. Identification of DEGs and Enrichment Evaluations

**Figure 3:**
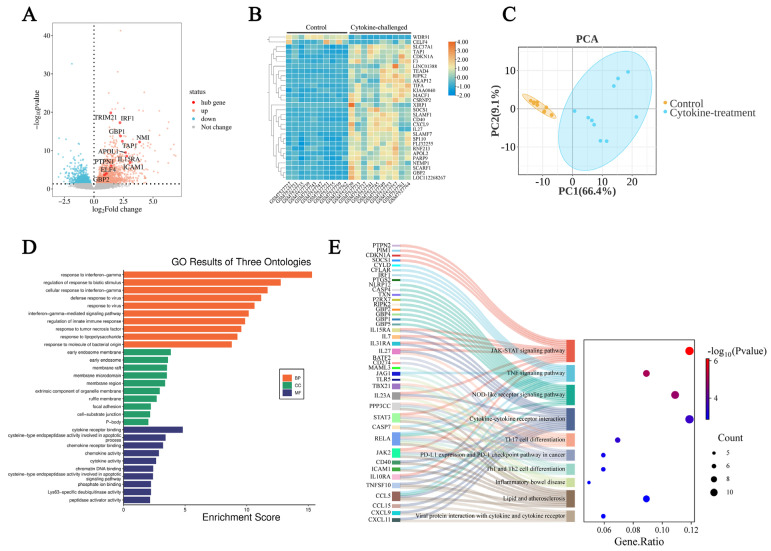
Identification of differentially expressed genes (DEGs) in the GSE236294 dataset. (**A**) A volcano plot of DEGs. Pink dots, upregulated genes; blue dots, downregulated genes; gray dots, genes with no significant difference in expression. (**B**) A heatmap of the top 30 DEGs. (**C**) A PCA plot of gene expression data. PC1 and PC2 explain 66.4% and 9.1% of the total variance, respectively. (**D**) Gene Ontology (GO) results of DEGs. All GO terms were grouped into three ontologies: orange, biological process (BP); green, cellular component (CC); and blue, molecular function (MF). (**E**) The distribution relationship between key genes and pathways displayed by a Sankey diagram. Among the top significant pathways, the gene *ICAM1* is closely associated with the TNF signaling pathway and the lipid and atherosclerosis pathway.

Before this analysis, GEO2R applied quantile normalization for data pretreatment, and the resulting boxplot from GSE236294 dataset analysis indicates that the normalization process has been well executed (Supplementary Figure S1). The gene expression signatures obtained from the GSE236294 dataset are presented in a volcano plot, which was inspected utilizing the GEO2R tool (Figure 3A). Based on the threshold of |logFC(fold change)| ≥ 1 plus adj.*p*-value < 0.05, 203 upregulated and 24 downregulated DEGs (Supplementary Table S4) were collected after manual inspection and used for further study. The heatmap of the top 30 DEGs, ranked by adjusted *p*-values, is shown in Figure 3B. Moreover, PCA indicated a distinct division between the control group and the group corresponding to macrophages challenged with cytokines (Figure 3C).

Functional enrichments were conducted to clarify the functions of DEGs. The top 10 terms for biological process (BP), cellular component (CC), and molecular function (MF) are presented in Figure 3D. The MF terms assigned to the DEGs were primarily associated with cytokine receptor binding, cysteine-type endopeptidase activity involved in apoptotic processes, and chemokine receptor binding. The possible CC terms involved included early endosome membrane, early endosome, and membrane raft. The BP terms of these genes were associated with response to interferon-gamma, regulation of response to biotic stimulus, and cellular response to interferon-gamma. In addition, the Sankey diagram visually represents the distribution of key genes in the different KEGG pathways, with the JAK-STAT, TNF, and NOD-like receptor signaling pathways ranking as the top three (Figure 3E).

### 2.3. PPI Assessment

**Figure 4:**
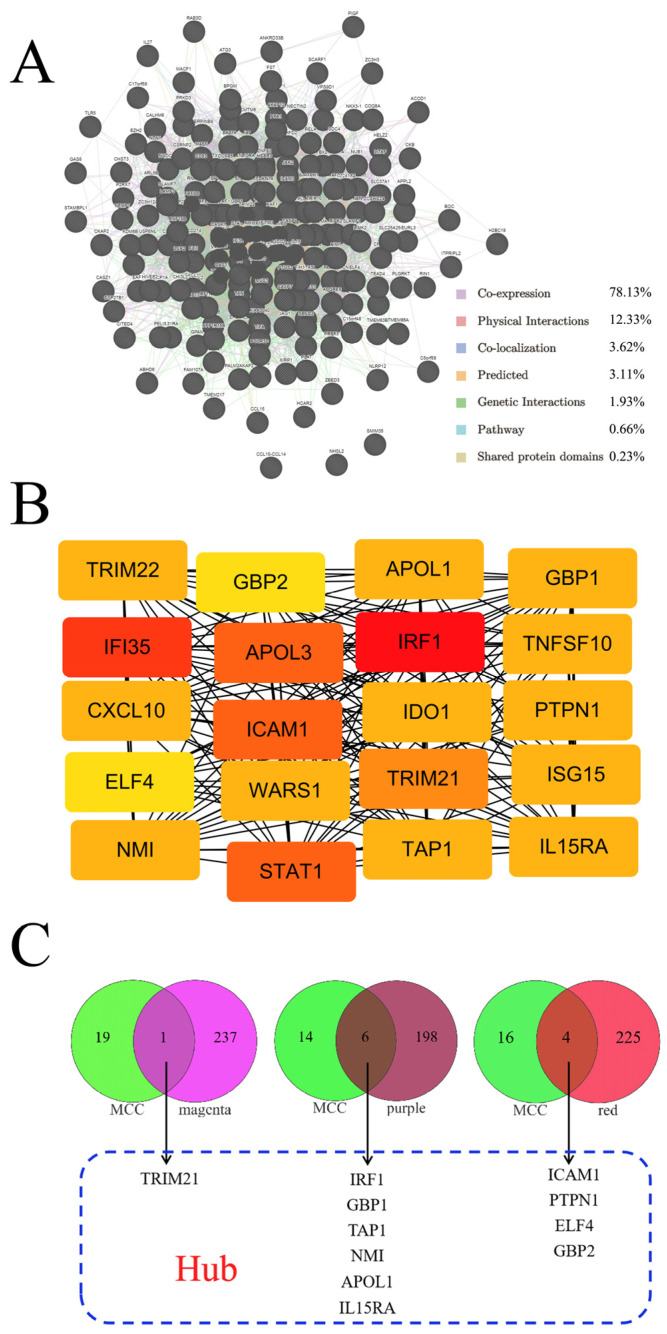
(**A**) Interaction networks of differentially expressed genes analyzed using GeneMANIA. Black circles, genes; colored lines, interactions between genes. Co-expression was the most common interaction type. (**B**) The top 20 key genes identified by MCC. Deeper red represents higher MCC scores. (**C**) The 11 hub genes according to the intersection of MCC with WGCNA.

Interaction networks identified by GeneMANIA depict a community of proteins or genes that share a similar function and are intimately connected by genetic and/or physical interactions [17]. The interaction networks identified for our DEGs were co-expression (78.13%), physical interactions (12.33%), co-localization (3.62%), predicted interactions (3.11%), genetic interactions (1.93%), pathway (0.66%), and sharing of protein domains (0.23%) (Figure 4A). Co-expression represented the most general interaction type in the DEG network (Figure 4A); these results suggest that the DEGs may perform similar functions or actions. Using the MCC methodology, the leading 20 key genes were identified, and the top three genes were *interferon regulatory factor 1* (*IRF1*), *interferon-induced protein 35* (*IFI35*), and *intercellular adhesion molecule 1* (*ICAM-1*) (Figure 4B and Supplementary Table S5).

### 2.4. Hub Gene Identification

**Figure 5:**
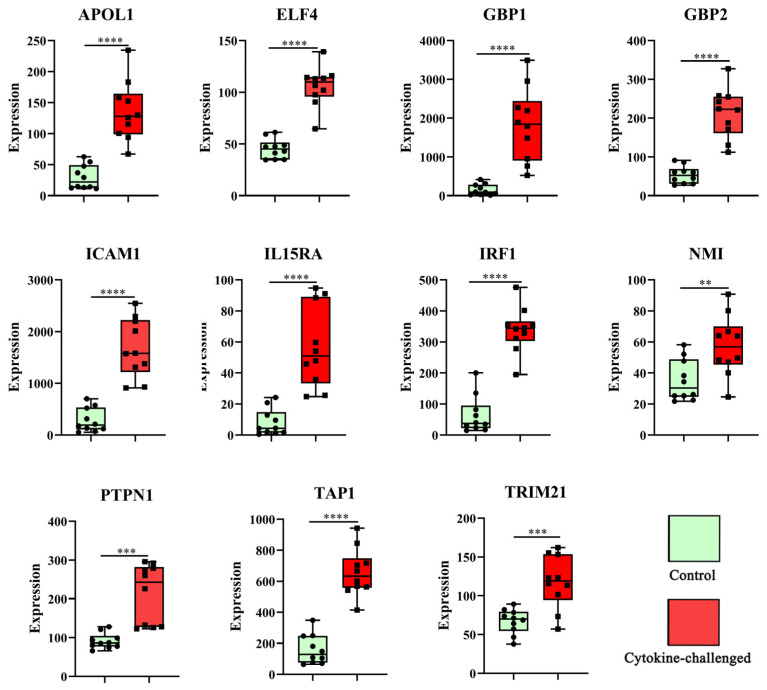
The relative expression of 11 hub genes in human monocyte-derived macrophages in the GSE236294 dataset. Green, control; red, cytokine-challenged. **, *p* < 0.01; ***, *p* < 0.001; ****, *p* < 0.0001.

**Figure 6:**
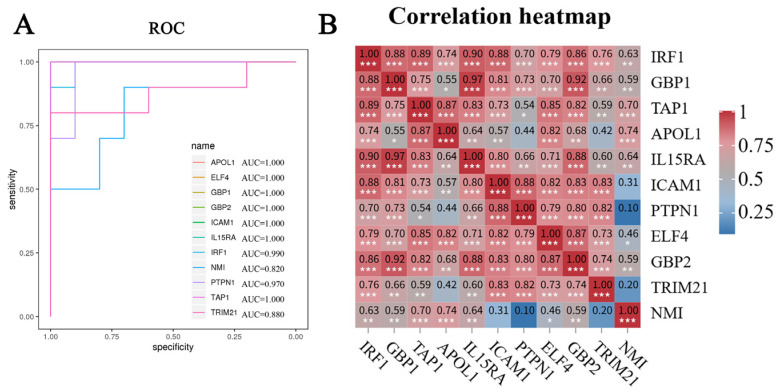
The AUC of the ROC curve (**A**) and the correlation heatmap (**B**) for the 11 hub genes in the GSE236294 dataset. *, *p* < 0.05; **, *p* < 0.01; ***, *p* < 0.001.

As shown in Figure 4C, 11 differential genes were derived from the overlap of the WCGNA results and MCC analyses, and they were considered the hub genes for macrophage-induced cytokine storm. The distribution of these top hubs is also depicted as a volcano plot in Figure 3A. As depicted in the expression maps (Figure 5), compared to the control group, the 11 hub genes were upregulated in cytokine-challenged groups. Furthermore, the ROC curve indicates that the calculated AUC values were all >0.80, implying that all hub genes possessed high clinical values (Figure 6A). Moreover, a Pearson correlation analysis of these genes is shown in Figure 6B.

### 2.5. Drug Prediction

The L1000CDS2 tool was used to probe small molecules that could reverse the identified gene expression signatures associated with macrophage-induced cytokine storm, and hence might have therapeutic potential. The top 50 compounds together with detailed information are described in Supplementary Table S6. The top three compounds that restored the gene signature were dasatinib (BRD-K49328571), vorinostat (BRD-K81418486), and metixene (BRD-A33711280) (Supplementary Table S6).

**Figure 7:**
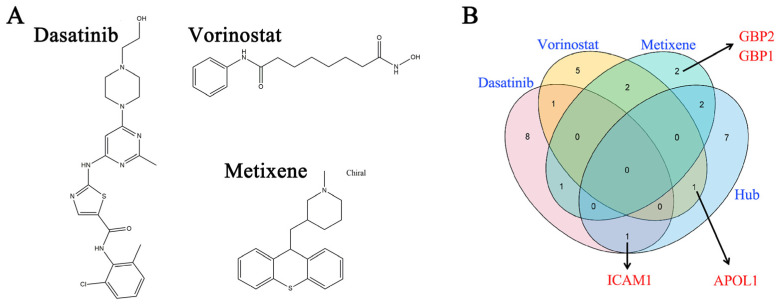
(**A**) The chemical structures of dasatinib, vorinostat, and metixene. (**B**) The overlap of cytokine storm-related differentially expressed genes with the gene signatures of drug candidate perturbation. ICAM1, APOL1, GBP1, and GBP2 were identified as hub genes from the intersection of the MCC and WGCNA results.

As summarized by Drugbank (Table 1), dasatinib is used for leukemia or chronic myeloid leukemia treatment, vorinostat is used to treat cutaneous T-cell lymphoma, and metixene is an anti-Parkinsonian agent. The chemical formulas of these three clinical drugs are shown in Figure 7A. A PubMed search (conducted on 8 October 2023) identified more than 20 research reports about the use of dasatinib in cytokine-related disease, contradictory results of the use of vorinostat for COVID-19, and almost no reports about the use of metixene for treating cytokine storm or COVID-19. Based on our results and the PubMed search, dastatinib might perturb key proteins related to COVID-19, and further research is warranted to investigate the repurposing of this clinically used drug.

**Figure 8:**
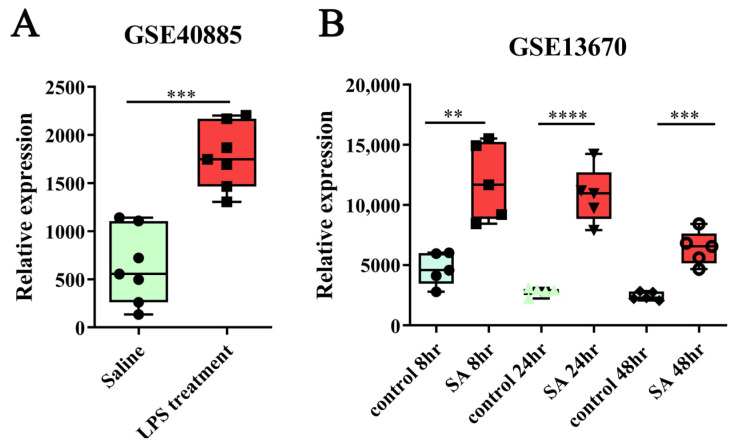
ICAM-1 relative expression in human macrophages from the Gene Expression Omnibus database. (**A**) GSE40885. Alveolar macrophages, saline (*n* = 7) vs. lipopolysaccharide (LPS) (*n* = 7). (**B**) GSE13670. Human monocyte-derived macrophages, control 8 h vs. SA 8 h; control 24 h vs. SA 24 h; control 48 h vs. SA 48 h (*n* = 5). SA: *Staphylococcus aureus*. **, *p* < 0.01; ***, *p* < 0.001; ****, *p* < 0.0001. *n =* number of samples per group.

Additionally, dasatinib, vorinostat, and metixene modulated different gene signatures (Table 2). By integrating the perturbed gene signatures with the hub results (Figure 7B), we identified that *ICAM-1* is a likely hub gene perturbed by dasatinib, *APOL1* is a likely hub gene perturbed by vorinostat, and *GBP1* and *GBP2* are likely hub genes perturbed by metixene (shown by the red label in Figure 7B). A search of the PubMed database (conducted on 8 October 2023) found no reports about the use of metixene in cytokine-related diseases. Therefore, ICAM-1 might be a key drug target for cytokine storm that is expressed in macrophages. To determine whether ICAM-1 exists in other disease-relevant situations, we analyzed relevant GEO raw data. We found that besides GSE236294 (Figure 5), *ICAM-1* was significantly upregulated in human monocyte-derived macrophages exposed to different cytokine challenges in the GSE40885 and GSE13670 datasets (Figure 8A,B).

### 2.6. Molecular Docking and Molecular Dynamic (MD) Verifications

**Figure 9:**
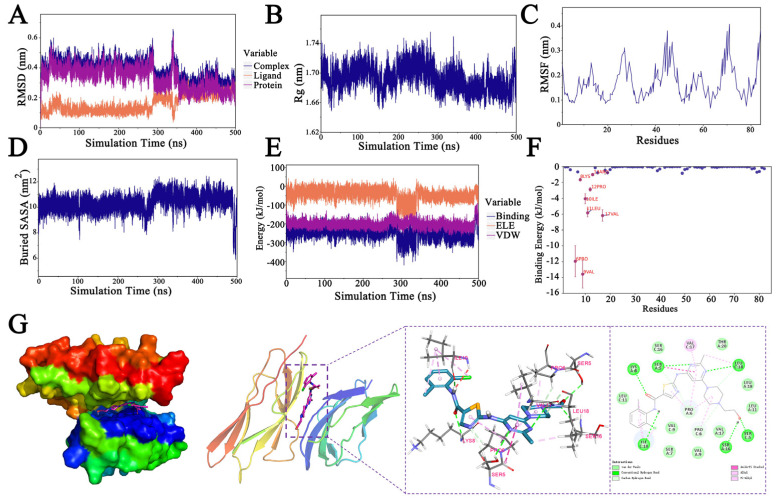
Molecular dynamics simulation of the dasatinib–ICAM-1 complex. (**A**) The backbone root mean square deviation (RMSD); (**B**) The radius of gyration (Rg); (**C**) The root mean square fluctuation (RMSF); (**D**) The buried solvent accessible surface area (SASA); (**E**) Energy; (**F**) Binding Energy; (**G**) The molecular docking model.

Dasatinib and ICAM-1 were selected for MD simulation analysis. The synchronous fluctuation of the root mean square deviation (RMSD) between the dasatinib–ICAM-1 complex and ICAM-1 suggests that the complex’s fluctuations stem from those in the protein structure, and as the simulation progresses, these fluctuations gradually decrease, indicating increased stability of the dasatinib-ICAM-1 complex (Figure 9A). The radius of gyration (Rg), a key metric reflecting molecular compactness, obtained by measuring the average atomic distance from the center of mass, gradually decreased from 1.8 nm for the dasatinib-ICAM-1 complex during simulation, indicating enhanced structural stability (Figure 9B). The root mean square fluctuation (RMSF) results and the buried Solvent Accessible Surface Area (SASA) collectively suggest the stability of the complex (Figure 9C,D). In addition, during the simulation, the binding energy of the complex, represented by the sum of VDW (van der Waals force and hydrophobic interactions) and ELE (electrostatic interaction), remained constant without considering solvation effects (Figure 9E). The complex exhibited an interaction energy of −117.946 ± 11.09 kcal/mol, signifying strong binding energy and affinity between dasatinib and ICAM-1, with negative binding free energy suggesting a spontaneous binding interaction (Table 3).

Hotspot residues refer to several residues that crucially contribute to the binding energy [18]. Key residues such as Val-9 and Pro-6 are known to significantly contribute to the corresponding binding energies within the complex (Figure 9F). As can more clearly be seen from the two-dimensional diagram of molecular docking interactions, the amino acids, including LYS-8, SER-5, LEU-18, SER-16, and ILE-10, form hydrogen bonds, while VAL-17, LEU-18, PRO-6, and ILE-10 engage in hydrophobic interactions, and THR-20, VAL-9, and LEU-11 interact via van der Waals forces with the small molecule (Figure 9G). Briefly, evaluations of RMSD, Rg, RMSF, and the binding metrics suggested that the dasatinib–ICAM-1 complex binds strongly and stably.

## 3. Discussion

Lung macrophages are pivotal actors in the cytokine storm triggered by COVID-19 [19]. The latest research has reported that the subpopulation of lung-resident alveolar macrophages (AMs), AM-Cd36, which exhibited the highest proportion of virus-positive cells at day 2 and active subgenomic transcription of SARS-CoV-2, started to decrease after day 2 and partially restored at day 14, indicating that SARS-CoV-2 infection damaged these AMs to escape lung innate immune defense [20]. In this study, the key modules and genes identified by the WGCNA and DEG results will aid the systematic analysis of gene interactions in macrophages challenged with cytokines.

GO analysis offers the most comprehensive method of understanding the functions of genes and gene products [21]. Our results show that the main BPs are mostly related to inflammatory response, playing a crucial function in the response to the virus (Figure 3D). Response to interferon-gamma was the first-ranked GO BP enriched in DEGs. A longitudinal cohort study reported that T-cell responses (i.e., IFNγ, IL2 and TNFα) were still highly cross-reactive in people who recovered from COVID-19 after 1 year [22] and 2 years [23]. Pathway enrichment analysis enables a deeper mechanistic understanding of genes derived from genome-scale (omics) experiments [24]. KEGG analysis revealed that the main pathways involved in inflammatory signaling pathways were the JAK-STAT signaling pathway, the TNF signaling pathway and the NOD-like receptor signaling pathway, among others (Figure 3E). These results help to more comprehensively understand the functions of DEGs.

As shown in Figure 4C, 11 biomarkers (TRIM21, IRF1, GBP1, TAP1, NMI, APOL1, IL15RA, ICAM-1, PTPN1, ELF4 and GBP2) were identified as the highest-ranked hub proteins. All these genes were significantly upregulated in macrophages challenged with cytokines (Figure 3A and Figure 5). Previous research identified 23 risk genes for severe COVID-19 [25], as well as 10 crucial genes—*ELANE*, *MPO*, *ARG1*, *DEFA4*, *CAMP*, *MMP9*, *LTF*, *LCN2*, *PGLYRP1*, and *HP*—that are commonly found in COVID-19 patients [26]. These findings enable us to better understand the genes involved in cytokine storm.

Silencing the cytokine storm in macrophages may be a potentially effective therapy for COVID-19. For example, baricitinib, a clinically applied JAK1/JAK2 agent, effectively alleviates macrophage inflammation and neutrophil infiltration in the low airways of rhesus macaques positive for SARS-CoV-2 [27]. Tubuloside B, extracted from Cistanche tubulosa, suppresses M1 macrophage activation [28]. A Syk kinase inhibitor modulates the immune-training of macrophages and SARS-CoV-2 infection [29]. Therefore, targeting macrophages to combat cytokine storm-related disease warrants further study.

L1000 is a powerful method to facilitate small-molecule discovery based on gene expression profiling [30]. The top 50 small-molecule compounds that reversed the gene expression signature of cytokine storm in macrophages were predicted by L1000CDS2 (Supplementary Table S6), and dasatinib, vorinostat, and metixene were the top-ranked drugs (Figure 7A, Table 1, Supplementary Table S6). These findings are consistent with previous research showing that dasatinib is likely one of the top 10 drug candidates for COVID-19 patients with benign prostatic hyperplasia [31]. Our findings are also in agreement with another report that identified vorinostat as a potential antiviral compound using Connectivity Map interrogation of a SARS-CoV-2 PPI network [32].

Dasatinib has been used in several drug repurposing studies for cytokine-related diseases. In animal or cell experiments, dasatinib (at concentrations above 50 nM) prevents abnormal cytokine release and cytotoxicity in activated T cells and prevents CD19-TCB-mediated B-cell depletion in humanized NSG mice [33]. Dasatinib pretreatment can temporarily inactivate CAR T cells, thereby mitigating acute cytokine toxicity and allowing T cells to regain their antitumor activities once the drug is withdrawn [34]. The combination of dasatinib and quercetin decreased SARS-CoV-2-induced mortality in mice [35], eliminated virus-induced senescence cells, mitigated COVID-19-reminiscent lung disease, and lessened inflammation in SARS-CoV-2-infected hamsters and mice [36]. In the clinic, dasatinib was used successfully to treat a patient with grade 3 cytokine release syndrome [37].

Vorinostat upregulates ACE2, which serves as the crucial receptor for SARS-CoV-2 entry, in various cell lines [38]. Vorinostat increases SARS-CoV-2 RNA abundance and enhances virus infection in cell models, conferring an apparent pro-viral effect [39]. However, computational analysis identified that vorinostat may be a promising drug candidate to mitigate the effects of SARS-CoV-2 [32,40]. Such inconsistent findings may be attributed to the cell-specific effects of the compound [39]. Moreover, dasatinib has been subjected to clinical trials for moderate and severe COVID-19 treatment (https://clinicaltrials.gov/study/NCT04830735, accessed on 23 March 2026) [41]. Therefore, of the three predicted drugs identified in this study (Table 1), dasatinib warrants further repurposing studies to determine its suitability for mitigating COVID-19-induced cytokine storm.

ICAM-1 was identified as a likely hub target of dasatinib (Figure 7B). ICAM-1 is an inducible cell adhesion protein of the immunoglobin family that mediates leukocyte adhesion to endothelial cells in inflammatory responses [42,43]. ICAM-1 has been hailed as a gatekeeper in various inflammatory diseases [42,43]. The plasma ICAM-1 concentration is statistically higher in non-survivors of COVID-19-related acute respiratory distress syndrome than survivors and is thus a predictor of mortality [44]. Serum ICAM-1 levels are elevated following recovery from COVID-19 infection, and so ICAM-1 levels may serve as a prognostic indicator for late clinical sequelae that result from COVID-19 [45]. ICAM-1 levels correlate with both CT radiological severity and other inflammatory markers in COVID-19 patients, suggesting that ICAM-1 is a reliable prognostic marker for COVID-19 [46]. All these studies are in agreement with our study, showing that ICAM-1 was upregulated in human monocyte-derived macrophages challenged by various inflammatory factors (Figure 8). Therefore, ICAM-1 has the potential to be a crucial therapeutic target for COVID-19-induced cytokine storm.

The findings suggest that dasatinib has various effects on ICAMs depending on the cell type and cellular signal pathways. For example, dasatinib treatment reduces ICAM-1 protein secretion from patient-derived endothelial cells [47]. In rats with pulmonary hypertension, chronic dasatinib treatment elevates levels of soluble ICAM-1, soluble VCAM-1, and soluble E-selectin [48]. Another report showed that dasatinib specifically decreases VCAM-1 expression, without affecting ICAM-1 and E-selectin expression in human aortic endothelial cells stimulated with TNF-α [49]. These findings indicate that dasatinib may directly act on ICAMs. Moreover, our molecular docking and MD results, which visualized interactions to gain a functional understanding of binding, support the notion that dasatinib and ICAM-1 spontaneously form a complex (Figure 9 and Table 3). Moreover, a recent study has reported that dasatinib attenuates betacoronavirus-induced inflammation and viral replication in macrophages through Src-MAPK pathway inhibition [50]. Thus, our findings indicate that dasatinib may represent a potential clinical candidate for treating cytokine storm elicited by macrophages through the targeting of ICAM-1.

Notably, as dasatinib is a tyrosine kinase inhibitor while ICAM-1 is an adhesion molecule lacking a canonical kinase domain, the potential direct interaction between dasatinib and ICAM-1 suggested by our MD analysis should be interpreted cautiously. Instead, ICAM-1 cross-linking activates Src tyrosine kinases via a signaling cascade requiring xanthine oxidase-derived reactive oxygen species and SHP-2 in human pulmonary microvascular endothelial cells [51]. In stromal fibroblasts and cancer-associated fibroblasts (CAFs), ICAM-1 regulates acto-myosin contractility and pro-invasive ECM remodeling via the Src/RhoA/ROCK/MLC2 signaling pathway [52]. Therefore, dasatinib may disrupt the ICAM-1–Src complex or bind to allosteric sites on ICAM-1 rather than a typical binding pocket. Further biophysical assays are needed to confirm direct or indirect bindings.

## 4. Materials and Methods

### 4.1. Data Capture

In the initial step, the keyword “macrophages” was used to search gene expression datasets based on the following inclusion criteria: (1) The samples must originate from the homo sapiens species. (2) The dataset must include both control and treatment groups. (3) Each group must have a sample size of at least five. (4) The datasets must provide raw data that can undergo reanalysis. (5) It is preferable that the data are related to lung tissue.

The GSE236294 datasets retrieved from the GEO database were subjected to a thorough analysis during this research (performed on 21 September 2023). The data were from primary cultures of human monocyte-derived macrophages that were challenged with pro-inflammatory cytokines to mimic the SARS-CoV-2-induced cytokine storm. In brief, 10 samples were treated with cytokines (IL1β, IL-6, IL8, TNFα, IFNγ and GM-CSF) at 20 ng/mL for 24 h; 10 samples were treated with vehicle (control RPMI plus 2% FBS). Samples were subject to microarray analysis in the GPL23227 platform. In addition, the raw data of GSE40885 and GSE13670 were retrieved to validate the expression of interesting genes in subsequent procedures.

### 4.2. Weighted Gene Co-Expression Network Analysis (WGCNA)

WGCNA represents an unsupervised analytical approach that enables researchers to uncover clusters (modules) of highly correlated genes and to obtain information about complex gene expression patterns [53]. The analysis involves several stages, including cluster analysis, network construction, module detection, calculation of topological properties, and gene significance and module membership identification [53]. In this study, the WGCNA plugin (V0.0.6.230118) within TBtools II (v2.303) software was utilized to build a gene co-expression network of genes in macrophages exposed to cytokines. First, as part of its built-in operations, data normalization was performed, and the top 8000 genes were retained for network construction. A scale-free topology criterion with an R^2^ cutoff of 0.8 was applied to determine the soft-thresholding power. Then, to detect the gene co-expression modules, we set a minimum module size of 30 genes and a cut-tree height of 0.25. Subsequently, module membership and gene significance scores were calculated to quantitatively assess the relevance of key modules and key genes.

### 4.3. Recognition of DEGs and Enrichment Analyses

GEO2R can explore DEGs within a gene expression dataset using the limma package of R (3.6.3) [54]. This method was employed as a first step (performed on 21 September 2023). Genes with logFC > 0 indicated those with higher expression in the experimental group versus the control group and were deemed as upregulated genes, whereas those with logFC < 0 were considered downregulated genes. The raw *p*-values were adjusted using the Benjamini–Hochberg (BH) procedure to control the false discovery rate (FDR). DEGs that exhibited a |logFC(fold change)| ≥ 1 and adj.*p*-value < 0.05 (BH-corrected) were judged as statistically meaningful. After a thorough manual inspection, the identified DEGs were treated as the gene expression signature for the model of cytokine storm in macrophages challenged with cytokines and used for further in-depth exploration.

Then, expression heatmap creation and principal component analysis (PCA) of the DEGs were carried out. Furthermore, DEGs were sent to a bioinformatics platform (https://www.bioinformatics.com.cn/) for Gene Ontology (GO) and KEGG pathway enrichment analysis, enabling us to annotate the DEGs in terms of function (conducted on 28 September 2023).

### 4.4. Protein–Protein Interactions (PPIs) and Hub Genes

PPIs offer a more comprehensive understanding of their biological functions and mechanisms [55]. The DEGs were uploaded to the GeneMANIA (http://genemania.org) online tool for network analysis, and *Homo sapiens* was chosen as the organism (conducted on 25 September 2023). This platform enables the identification of genes that work together and predicts their functions within the provided dataset by leveraging both genomic and proteomic data [56].

Hubs refer to highly connected and functionally essential proteins that have important roles in maintaining the network’s structure and function [57]. Maximal clique centrality (MCC) is a newly proposed method to identify essential central hub proteins in a biological network that outperforms 11 other algorithms in terms of accuracy [58]. During this procedure, the PPI network for DEGs was built utilizing the GeneMANIA results obtained in the previous step. Subsequently, the crucial genes within the PPI network were evaluated by using the maximal clique centrality (MCC) method in the CytoHubba plugin of Cytoscape 3.7.1 software (performed on 25 September 2023); the 20 top-scoring genes were identified for the next step of the study.

Finally, we intersected genes derived from WCGNA and MCC analyses. The overlapping genes were identified as potential hub genes that are closely linked with macrophage-induced cytokine storm. The relative expressions, the area under the ROC curve (AUC) and gene correlation of the hub genes were all determined based on the GSE236294 dataset.

### 4.5. Drug Predictions

The Connectivity Map is a systematic resource that uses gene expression signatures derived from cultured human cells exposed to biologically active small molecules to link drugs, genes, and diseases [59]. L1000 is a representative platform that contains 1058 probes for 978 landmark transcripts and 80 control transcripts to predict the expression of 81% of non-measured transcripts [30]. It also includes 1,319,138 gene expression profiles, encompassing 42,080 genetic and small-molecule perturbations that have been profiled across a wide variety of cell types [30]. Thus, the L1000 framework is an exceptional method for exploring the mode of action of small molecules, providing functional annotations for pathogenic-related genetic variants, and searching for potential strategies that interfere with gene networks [30].

To identify drugs that might be linked to the identified gene expression profiles using the Connectivity Map, the DEGs were submitted to the L1000CDS2 platform (https://maayanlab.cloud/L1000CDS2/#/index, accessed on 12 October 2023), which relies on the L1000 database (performed on 1 October 2023) [60]. Significantly upregulated and downregulated DEGs were input into the corresponding up- and downregulated gene fields, respectively, with gene symbols used as the gene identifier. Thus, according to their overlap scores, the top 50 chemical molecules that could normalize the expression profile of interest were ranked and used for in-depth examination.

### 4.6. Molecular Docking and Molecular Dynamics (MD) Studies

Protein intercellular adhesion molecule 1 (ICAM-1) and clinically used dasatinib were identified for further investigation. Briefly, the crystalline structure of ICAM-1 (PDB: 1MQ8) was downloaded from the PDB database (https://www.rcsb.org/); The dasatinib structure file (CID: 3062316) was obtained from the PubChem database. Molecular docking was performed with AutoDock Vina (1.2.5) software, while MD simulations (500 ns) were conducted using the Gromacs 2022 program. The procedure and parameter configurations utilized in this step were similar to those used in previous research [61]. Briefly, amber14SB/TIP3P was used for the protein/solvent, and the ligand was parameterized with Antechamber (AM1-BCC, GAFF2) and converted by ACPYPE with TIP3P-compatible Joung–Cheatham ions. The complex was solvated in a truncated dodecahedral box (≥1.2 nm), neutralized and set to 0.15 M NaCl, minimized (SD, Fmax < 1000 kJ·mol^−1^·nm^−1^), and equilibrated (298 K; 200 ps NVT then NPT). The production run was conducted under NPT conditions for 500 ns with a 2 fs time step and Verlet cutoff; Coulomb interactions were calculated via PME, and both van der Waals and Coulomb cutoffs were 1.2 nm. All hydrogen bonds were constrained by LINCS. Temperature and pressure were maintained at 298 K and 1 bar using Nosé–Hoover and Parrinello–Rahman methods, respectively; frames were saved every 10 ps and analyzed in GROMACS(2022)/VMD(1.9.3)/PyMOL(2.62), with MM-PBSA (gmx_MMPBSA) as needed.

## 5. Summary

The overall objective of this study was to assess gene expression, identify central genes, and find potential therapeutic agents for cytokine storm resulting from macrophage overactivation. We initially identified the key gene modules and key genes in the GSE236294 dataset. This dataset was derived from primary cultured human monocyte-derived macrophages treated with cytokines. Secondly, we observed 203 upregulated and 24 downregulated DEGs; Then, PPI and hub analyses revealed that *TRIM21*, *IRF1*, *GBP1*, *TAP1*, *NMI*, *APOL1*, *ICAM1*, *PTPN1*, *ELF4*, and *GBP2* are likely hub genes. Furthermore, L1000CDS2 analysis of the gene expression signatures suggested that dasatinib, a clinically used drug for treating leukemia, may reverse ICAM-1 expression and have the potential to treat cytokine storm-related disease. Moreover, molecular docking and MD results revealed that the dasatinib–ICAM-1 complex spontaneously forms and is relatively stable. In short, dasatinib might represent a potential clinical drug candidate for the treatment of macrophage-induced cytokine storm in humans, likely by targeting ICAM-1. Nevertheless, due to limitations in bioinformatics studies, additional wet-lab studies are required to validate these findings. Moreover, since this study models inflammation using cytokine-stimulated macrophages rather than direct SARS-CoV-2 infection, these findings reflect general macrophage inflammatory responses and must be confirmed in viral infection models before extrapolation to COVID-19.

## Figures and Tables

**Table 1:** Information about the three drug candidates.

Perturbation	Drugbank	Summary
Dasatinib	DB01254	A tyrosine kinase inhibitor used for the treatment of Philadelphia chromosome-positive acute lymphoblastic leukemia or chronic myeloid leukemia.
Vorinostat	DB02546	A histone deacetylase (HDAC) inhibitor used for the treatment of cutaneous manifestations in patients with progressive, persistent, or recurrent cutaneous T-cell lymphoma (CTCL) following prior systemic therapies.
Metixene hydrochloride	DB00340	An anticholinergic used as an anti-parkinsonian agent.

**Table 2:** The overlap of cytokine storm-related differentially expressed genes with the gene signatures of drug candidate perturbation.

Drug	Input Up/Signature Down	Input Down/Signature Up
dasatinib	*DUSP5*, *F3*, *FST*, *ICAM-1*, *MAFF*, *PIM1*, *PLAT*, *PTGS2*, *SDC4*, *TXN*, *ZC3H12A*	None
vorinostat	*APOL1*, *CCL5*, *CXCL11*, *CXCL9*, *MAFF*, *MTHFD2*, *NBN*, *RCN1*, *RHOQ*	None
Metixene	*CD40*, *CXCL11*, *CXCL9*, *FST*, *GBP1*, *GBP2*, *TNFSF10*	None

Note: Input up/signature down indicates that drugs downregulate disease-upregulated genes, while input down/signature up shows the opposite pattern.

**Table 3:** The binding energy (kal/mol) of the dasatinib–ICAM-1 complex.

Molecular Interaction	Binding Energy (kcal/mol)
ΔEvdw (Van der Waals Interaction)	−159.069 ± 16.747
ΔEele (Electrostatic Interaction)	−6.599 ± 1.282
ΔEpol (Polar Solvation Energy)	70.238 ± 8.716
ΔEnonpol (Nonpolar Solvation Energy)	−22.517 ± 1.977
ΔEMMPBSA (ΔEMMPBSA = ΔEvdw + ΔEele + ΔEpol + ΔEnonpol)	−117.946 ± 11.09

## Data Availability

The raw data from GSE236294, GSE40885, and GSE13670 are freely downloaded from the GEO database. Other raw data are available from the corresponding author.

## Supplementary Materials

The following supporting information can be downloaded at: https://www.mdpi.com/article/10.3390/molecules31071111/s1, Figure S1: GSE236294 dataset normalization boxplot. Table S1: Hub genes of magenta modules. Table S2: Hub genes of purple modules. Table S3: Hub genes of red modules. Table S4: Differentially expressed genes (DEGs) of GSE236294 dataset. Table S5: Top 20 in network genemania-interactions.txt ranked by MCC method. Table S6: The potential small compounds identified by L1000CDS^2^.

## References

[1] Zarocostas J. (2023). With the COVID-19 PHEIC over, what next?. Lancet.

[2] Maity S., Santra A., Hebbani A.V., Pulakuntla S., Chatterjee A., Badri K.R., Damodara Reddy V. (2023). Targeting cytokine storm as the potential anti-viral therapy: Implications in regulating SARS-CoV-2 pathogenicity. Gene.

[3] Yang L., Xie X., Tu Z., Fu J., Xu D., Zhou Y. (2021). The signal pathways and treatment of cytokine storm in COVID-19. Signal Transduct. Target. Ther..

[4] Huang C., Wang Y., Li X., Ren L., Zhao J., Hu Y., Zhang L., Fan G., Xu J., Gu X. (2020). Clinical features of patients infected with 2019 novel coronavirus in Wuhan, China. Lancet.

[5] Mangalmurti N., Hunter C.A. (2020). Cytokine Storms: Understanding COVID-19. Immunity.

[6] Merad M., Martin J.C. (2020). Pathological inflammation in patients with COVID-19: A key role for monocytes and macrophages. Nat. Rev. Immunol..

[7] Manjili R.H., Zarei M., Habibi M., Manjili M.H. (2020). COVID-19 as an Acute Inflammatory Disease. J. Immunol..

[8] Ng W.H., Tang P.C.H., Mahalingam S., Liu X. (2023). Repurposing of drugs targeting the cytokine storm induced by SARS-CoV-2. Br. J. Pharmacol..

[9] Li M., Wang M., Wen Y., Zhang H., Zhao G.N., Gao Q. (2023). Signaling pathways in macrophages: Molecular mechanisms and therapeutic targets. MedComm.

[10] Felkle D., Zieba K., Kaleta K., Czaja J., Zyzdorf A., Sobocinska W., Jarczynski M., Bryniarski K., Nazimek K. (2023). Overreactive macrophages in SARS-CoV-2 infection: The effects of ACEI. Int. Immunopharmacol..

[11] Brautigam K., Reinhard S., Wartenberg M., Forster S., Greif K., Granai M., Bosmuller H., Klingel K., Schurch C.M. (2023). Comprehensive analysis of SARS-CoV-2 receptor proteins in human respiratory tissues identifies alveolar macrophages as potential virus entry site. Histopathology.

[12] Wang C., Xie J., Zhao L., Fei X., Zhang H., Tan Y., Nie X., Zhou L., Liu Z., Ren Y. (2020). Alveolar macrophage dysfunction and cytokine storm in the pathogenesis of two severe COVID-19 patients. EBioMedicine.

[13] Delorey T.M., Ziegler C.G.K., Heimberg G., Normand R., Yang Y., Segerstolpe A., Abbondanza D., Fleming S.J., Subramanian A., Montoro D.T. (2021). COVID-19 tissue atlases reveal SARS-CoV-2 pathology and cellular targets. Nature.

[14] Laurent P., Yang C., Rendeiro A.F., Nilsson-Payant B.E., Carrau L., Chandar V., Bram Y., tenOever B.R., Elemento O., Ivashkiv L.B. (2022). Sensing of SARS-CoV-2 by pDCs and their subsequent production of IFN-I contribute to macrophage-induced cytokine storm during COVID-19. Sci. Immunol..

[15] Liao M., Liu Y., Yuan J., Wen Y., Xu G., Zhao J., Cheng L., Li J., Wang X., Wang F. (2020). Single-cell landscape of bronchoalveolar immune cells in patients with COVID-19. Nat. Med..

[16] Abdelmoaty M.M., Yeapuri P., Machhi J., Olson K.E., Shahjin F., Kumar V., Zhou Y., Liang J., Pandey K., Acharya A. (2021). Defining the Innate Immune Responses for SARS-CoV-2-Human Macrophage Interactions. Front. Immunol..

[17] Vella D., Zoppis I., Mauri G., Mauri P., Di Silvestre D. (2017). From protein-protein interactions to protein co-expression networks: A new perspective to evaluate large-scale proteomic data. EURASIP J. Bioinform. Syst. Biol..

[18] Zhang X., Wang Y., Ji J., Si D., Bao X., Yu Z., Zhu Y., Zhao L., Li W., Liu J. (2022). Discovery of 1,6-Naphthyridin-2(1H)-one Derivatives as Novel, Potent, and Selective FGFR4 Inhibitors for the Treatment of Hepatocellular Carcinoma. J. Med. Chem..

[19] Olivier T., Blomet J., Desmecht D. (2023). Central role of lung macrophages in SARS-CoV-2 physiopathology: A cross-model single-cell RNA-seq perspective. Front. Immunol..

[20] Cong B., Dong X., Yang Z., Yu P., Chai Y., Liu J., Zhang M., Zang Y., Kang J., Feng Y. (2024). Single-cell spatiotemporal analysis of the lungs reveals Slamf9^+^ macrophages involved in viral clearance and inflammation resolution. Cell Discov..

[21] Gene Ontology Consortium (2021). The Gene Ontology resource: Enriching a GOld mine. Nucleic Acids Res..

[22] Guo L., Wang G., Wang Y., Zhang Q., Ren L., Gu X., Huang T., Zhong J., Wang Y., Wang X. (2022). SARS-CoV-2-specific antibody and T-cell responses 1 year after infection in people recovered from COVID-19: A longitudinal cohort study. Lancet Microbe.

[23] Guo L., Zhang Q., Gu X., Ren L., Huang T., Li Y., Zhang H., Liu Y., Zhong J., Wang X. (2023). Durability and cross-reactive immune memory to SARS-CoV-2 in individuals 2 years after recovery from COVID-19: A longitudinal cohort study. Lancet Microbe.

[24] Reimand J., Isserlin R., Voisin V., Kucera M., Tannus-Lopes C., Rostamianfar A., Wadi L., Meyer M., Wong J., Xu C. (2019). Pathway enrichment analysis and visualization of omics data using g:Profiler, GSEA, Cytoscape and EnrichmentMap. Nat. Protoc..

[25] Qi H.X., Shen Q.D., Zhao H.Y., Qi G.Z., Gao L. (2022). Network-based analysis revealed significant interactions between risk genes of severe COVID-19 and host genes interacted with SARS-CoV-2 proteins. Brief. Bioinform..

[26] Ramesh P., Veerappapillai S., Karuppasamy R. (2021). Gene expression profiling of corona virus microarray datasets to identify crucial targets in COVID-19 patients. Gene Rep..

[27] Hoang T.N., Pino M., Boddapati A.K., Viox E.G., Starke C.E., Upadhyay A.A., Gumber S., Nekorchuk M., Busman-Sahay K., Strongin Z. (2021). Baricitinib treatment resolves lower-airway macrophage inflammation and neutrophil recruitment in SARS-CoV-2-infected rhesus macaques. Cell.

[28] Xiao L., Yao J., Miao Y., Ou B., Wang J., Huang Y., Zhou B., Ge L., Tian J., Zeng X. (2022). Tubuloside B, isolated from Cistanche tubulosa, a promising agent against M1 macrophage activation via synergistically targeting Mob1 and ERK1/2. Biomed. Pharmacother..

[29] John S.P., Singh A., Sun J., Pierre M.J., Alsalih L., Lipsey C., Traore Z., Balcom-Luker S., Bradfield C.J., Song J. (2022). Small-molecule screening identifies Syk kinase inhibition and rutaecarpine as modulators of macrophage training and SARS-CoV-2 infection. Cell Rep..

[30] Subramanian A., Narayan R., Corsello S.M., Peck D.D., Natoli T.E., Lu X., Gould J., Davis J.F., Tubelli A.A., Asiedu J.K. (2017). A Next Generation Connectivity Map: L1000 Platform and the First 1,000,000 Profiles. Cell.

[31] Zhou H., Xu M., Hu P., Li Y., Ren C., Li M., Pan Y., Wang S., Liu X. (2023). Identifying hub genes and common biological pathways between COVID-19 and benign prostatic hyperplasia by machine learning algorithms. Front. Immunol..

[32] Adhami M., Sadeghi B., Rezapour A., Haghdoost A.A., MotieGhader H. (2021). Repurposing novel therapeutic candidate drugs for coronavirus disease-19 based on protein-protein interaction network analysis. BMC Biotechnol..

[33] Leclercq G., Haegel H., Schneider A., Giusti A.M., Marrer-Berger E., Boetsch C., Walz A.C., Pulko V., Sam J., Challier J. (2021). Src/lck inhibitor dasatinib reversibly switches off cytokine release and T cell cytotoxicity following stimulation with T cell bispecific antibodies. J. Immunother. Cancer.

[34] Mestermann K., Giavridis T., Weber J., Rydzek J., Frenz S., Nerreter T., Mades A., Sadelain M., Einsele H., Hudecek M. (2019). The tyrosine kinase inhibitor dasatinib acts as a pharmacologic on/off switch for CAR T cells. Sci. Transl. Med..

[35] Pastor-Fernandez A., Bertos A.R., Sierra-Ramirez A., Del Moral-Salmoral J., Merino J., de Avila A.I., Olague C., Villares R., Gonzalez-Aseguinolaza G., Rodriguez M.A. (2023). Treatment with the senolytics dasatinib/quercetin reduces SARS-CoV-2-related mortality in mice. Aging Cell.

[36] Lee S., Yu Y., Trimpert J., Benthani F., Mairhofer M., Richter-Pechanska P., Wyler E., Belenki D., Kaltenbrunner S., Pammer M. (2021). Virus-induced senescence is a driver and therapeutic target in COVID-19. Nature.

[37] Baur K., Heim D., Beerlage A., Poerings A.S., Kopp B., Medinger M., Dirks J.C., Passweg J.R., Holbro A. (2022). Dasatinib for treatment of CAR T-cell therapy-related complications. J. Immunother. Cancer.

[38] Sinha S., Cheng K., Schaffer A.A., Aldape K., Schiff E., Ruppin E. (2020). In vitro and in vivo identification of clinically approved drugs that modify ACE2 expression. Mol. Syst. Biol..

[39] Ravindran V., Wagoner J., Athanasiadis P., Den Hartigh A.B., Sidorova J.M., Ianevski A., Fink S.L., Frigessi A., White J., Polyak S.J. (2022). Discovery of host-directed modulators of virus infection by probing the SARS-CoV-2-host protein-protein interaction network. Brief. Bioinform..

[40] Tomazou M., Bourdakou M.M., Minadakis G., Zachariou M., Oulas A., Karatzas E., Loizidou E.M., Kakouri A.C., Christodoulou C.C., Savva K. (2021). Multi-omics data integration and network-based analysis drives a multiplex drug repurposing approach to a shortlist of candidate drugs against COVID-19. Brief. Bioinform..

[41] Schake P., Dishnica K., Kaiser F., Leberecht C., Haupt V.J., Schroeder M. (2023). An interaction-based drug discovery screen explains known SARS-CoV-2 inhibitors and predicts new compound scaffolds. Sci. Rep..

[42] Singh V., Kaur R., Kumari P., Pasricha C., Singh R. (2023). ICAM-1 and VCAM-1: Gatekeepers in various inflammatory and cardiovascular disorders. Clin. Chim. Acta.

[43] Singh M., Thakur M., Mishra M., Yadav M., Vibhuti R., Menon A.M., Nagda G., Dwivedi V.P., Dakal T.C., Yadav V. (2021). Gene regulation of intracellular adhesion molecule-1 (ICAM-1): A molecule with multiple functions. Immunol. Lett..

[44] Spadaro S., Fogagnolo A., Campo G., Zucchetti O., Verri M., Ottaviani I., Tunstall T., Grasso S., Scaramuzzo V., Murgolo F. (2021). Markers of endothelial and epithelial pulmonary injury in mechanically ventilated COVID-19 ICU patients. Crit. Care.

[45] Smith-Norowitz T.A., Loeffler J., Norowitz Y.M., Kohlhoff S. (2021). Intracellular Adhesion Molecule-1 (ICAM-1) Levels in Convalescent COVID-19 Serum: A Case Report. Ann. Clin. Lab. Sci..

[46] Hamza A.M., Ali W.D.K., Hassanein N., Albassam W.B., Barry M., AlFaifi A.M.M., Altayyar K.A.S., Aboabat N.A.M., Alshaiddi W.K.F., AbuSabbah H.M.H. (2022). Relation between macrophage inflammatory protein-1 and intercellular adhesion molecule-1 and computed tomography findings in critically-ill saudi covid-19 patients. J. Infect. Public Health.

[47] de Jesus A.A., Chen G., Yang D., Brdicka T., Ruth N.M., Bennin D., Cebecauerova D., Malcova H., Freeman H., Martin N. (2023). Constitutively active Lyn kinase causes a cutaneous small vessel vasculitis and liver fibrosis syndrome. Nat. Commun..

[48] Guignabert C., Phan C., Seferian A., Huertas A., Tu L., Thuillet R., Sattler C., Le Hiress M., Tamura Y., Jutant E.M. (2016). Dasatinib induces lung vascular toxicity and predisposes to pulmonary hypertension. J. Clin. Investig..

[49] Murphy J.M., Jeong K., Rodriguez Y.A.R., Kim J.H., Ahn E.E., Lim S.S. (2019). FAK and Pyk2 activity promote TNF-alpha and IL-1beta-mediated pro-inflammatory gene expression and vascular inflammation. Sci. Rep..

[50] Ricoy A.C.S., Braga M.P., Lacerda T.T.F., Martins F.R.B., Mendes A.C., Teixeira M.M., Costa V.V., Bahia D., Soriani F.M. (2025). Dasatinib inhibits betacoronavirus replication in macrophages and attenuates pro-inflammatory mediators via SRC-MAPK pathway modulation. Med. Microbiol. Immunol..

[51] Wang Q., Pfeiffer G.R., Gaarde W.A. (2003). Activation of SRC tyrosine kinases in response to ICAM-1 ligation in pulmonary microvascular endothelial cells. J. Biol. Chem..

[52] Bonan S., Albrengues J., Grasset E., Kuzet S.E., Nottet N., Bourget I., Bertero T., Mari B., Meneguzzi G., Gaggioli C. (2017). Membrane-bound ICAM-1 contributes to the onset of proinvasive tumor stroma by controlling acto-myosin contractility in carcinoma-associated fibroblasts. Oncotarget.

[53] Langfelder P., Horvath S. (2008). WGCNA: An R package for weighted correlation network analysis. BMC Bioinform..

[54] Clough E., Barrett T., Wilhite S.E., Ledoux P., Evangelista C., Kim I.F., Tomashevsky M., Marshall K.A., Phillippy K.H., Sherman P.M. (2024). NCBI GEO: Archive for gene expression and epigenomics data sets: 23-year update. Nucleic Acids Res..

[55] Wang S., Wu R., Lu J., Jiang Y., Huang T., Cai Y.D. (2022). Protein-protein interaction networks as miners of biological discovery. Proteomics.

[56] Franz M., Rodriguez H., Lopes C., Zuberi K., Montojo J., Bader G.D., Morris Q. (2018). GeneMANIA update 2018. Nucleic Acids Res..

[57] Nithya C., Kiran M., Nagarajaram H.A. (2024). Hubs and Bottlenecks in Protein-Protein Interaction Networks. Methods Mol. Biol..

[58] Chin C.H., Chen S.H., Wu H.H., Ho C.W., Ko M.T., Lin C.Y. (2014). cytoHubba: Identifying hub objects and sub-networks from complex interactome. BMC Syst. Biol..

[59] Lamb J., Crawford E.D., Peck D., Modell J.W., Blat I.C., Wrobel M.J., Lerner J., Brunet J.P., Subramanian A., Ross K.N. (2006). The Connectivity Map: Using gene-expression signatures to connect small molecules, genes, and disease. Science.

[60] Duan Q., Reid S.P., Clark N.R., Wang Z., Fernandez N.F., Rouillard A.D., Readhead B., Tritsch S.R., Hodos R., Hafner M. (2016). L1000CDS^2^: LINCS L1000 characteristic direction signatures search engine. npj Syst. Biol. Appl..

[61] Wang X., Jia L., Xie Y., He T., Wang S., Jin X., Xie F. (2024). Deciphering the interaction mechanism between soy protein isolate and fat-soluble anthocyanin on experiments and molecular simulations. Int. J. Biol. Macromol..

