# Supplementary material for: Identifying ICAM-1 as a Therapeutic Target for Cytokine Storm in Human Macrophages Through Integrative Bioinformatics Approaches
Source: Molecules. 2026 Mar 27;31(7):1111. doi: 10.3390/molecules31071111 (PMC13075147; doi:10.3390/molecules31071111)
Supplement: Supplementary file 1 [file molecules-31-01111-s001.zip › molecules-4174001-supplementary/Supplementary Figure and Table Legends.pdf]

Supplementary Figure and Table Legends

Supplementary Figure S1: GSE236294 dataset normalization boxplot.

Supplementary Table S1: Hub genes of magenta modules.

Supplementary Table S2: Hub genes of purple modules.

Supplementary Table S3: Hub genes of red modules.

Supplementary Table S4: Differentially expressed genes (DEGs) of GSE236294 dataset.

Supplementary Table S5: Top 20 in network genemania-interactions.txt ranked by MCC method.

Supplementary Table S6: The potential small compounds identified by L1000CDS^2.
