# Supplementary figures and images for: Identifying ICAM-1 as a Therapeutic Target for Cytokine Storm in Human Macrophages Through Integrative Bioinformatics Approaches
Source: Molecules. 2026 Mar 27;31(7):1111. doi: 10.3390/molecules31071111 (PMC13075147; doi:10.3390/molecules31071111)

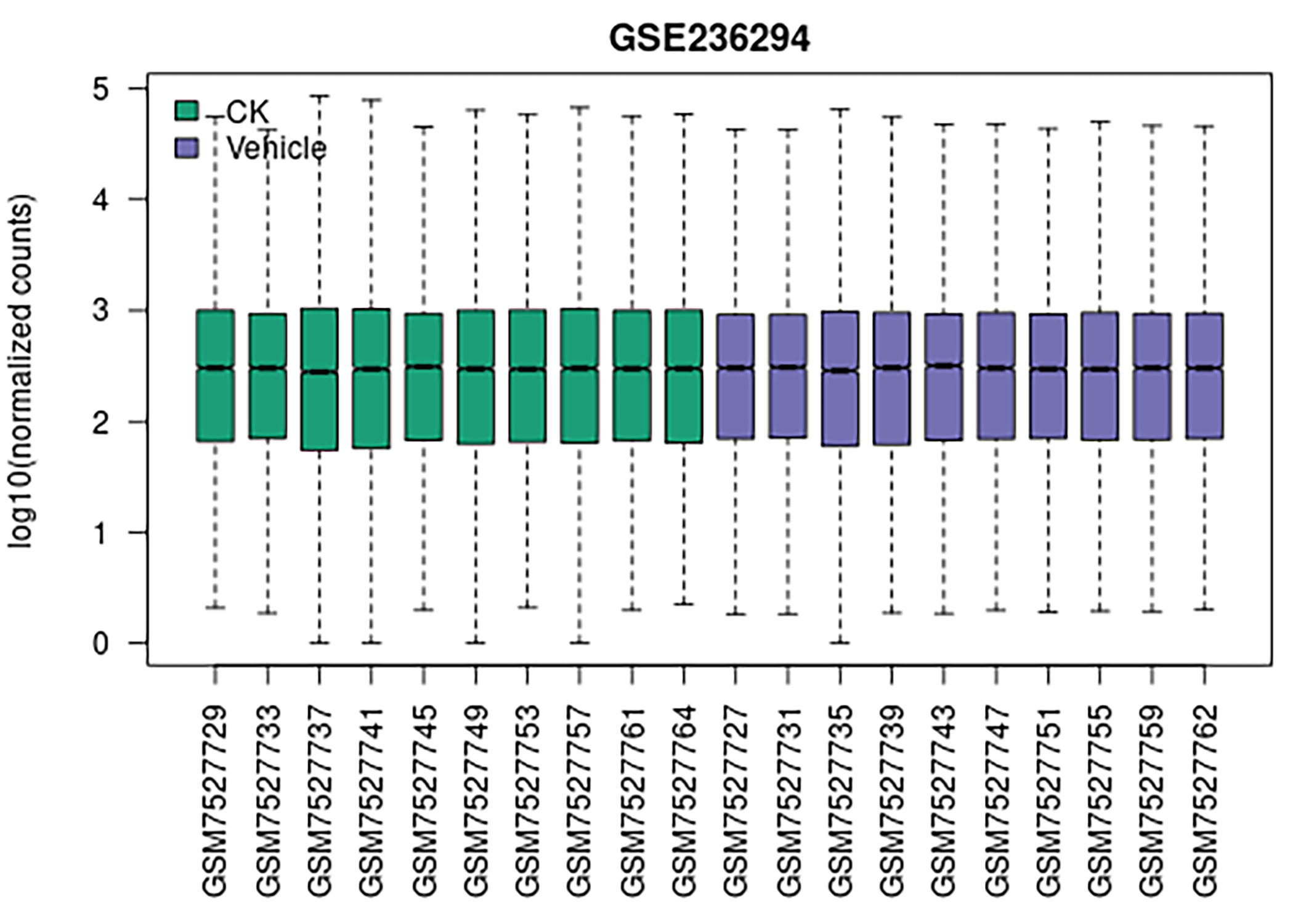

Supplement: Supplementary file 1 [file molecules-31-01111-s001.zip › molecules-4174001-supplementary/Supplementary Figure S1.tif]
